# Object Detection and Classification by Decision-Level Fusion for Intelligent Vehicle Systems

**DOI:** 10.3390/s17010207

**Published:** 2017-01-22

**Authors:** Sang-Il Oh, Hang-Bong Kang

**Affiliations:** Department of Media Engineering, Catholic University of Korea, 43-1, Yeoggok 2-dong, Wonmmi-gu, Bucheon-si, Gyeonggi-do 14662, Korea; nicolas0@catholic.ac.kr

**Keywords:** multiple sensor fusion, decision level fusion, object detection, object classification, object recognition, LiDAR, CCD

## Abstract

To understand driving environments effectively, it is important to achieve accurate detection and classification of objects detected by sensor-based intelligent vehicle systems, which are significantly important tasks. Object detection is performed for the localization of objects, whereas object classification recognizes object classes from detected object regions. For accurate object detection and classification, fusing multiple sensor information into a key component of the representation and perception processes is necessary. In this paper, we propose a new object-detection and classification method using decision-level fusion. We fuse the classification outputs from independent unary classifiers, such as 3D point clouds and image data using a convolutional neural network (CNN). The unary classifiers for the two sensors are the CNN with five layers, which use more than two pre-trained convolutional layers to consider local to global features as data representation. To represent data using convolutional layers, we apply region of interest (ROI) pooling to the outputs of each layer on the object candidate regions generated using object proposal generation to realize color flattening and semantic grouping for charge-coupled device and Light Detection And Ranging (LiDAR) sensors. We evaluate our proposed method on a KITTI benchmark dataset to detect and classify three object classes: cars, pedestrians and cyclists. The evaluation results show that the proposed method achieves better performance than the previous methods. Our proposed method extracted approximately 500 proposals on a 1226×370 image, whereas the original selective search method extracted approximately $10^{6}\times n$ proposals. We obtained classification performance with 77.72% mean average precision over the entirety of the classes in the moderate detection level of the KITTI benchmark dataset.

## 1. Introduction

Advanced driver assistant systems (ADASs) are utilized to assist drivers in unpredictable driving situations. ADASs can be classified into recognition systems and interfaces for warning alarm. Examples of the former include collision prediction and attention-less driver detection. The second one includes providing information to drivers about hazardous events. However, an essential task in both types of ADASs is reliable detection of other objects and events, known as simultaneous localization, mapping and moving-object tracking [1,2,3], or the detection and tracking of moving objects.

Effective object detection and classification methods that localize and identify objects of interest are very important in many fields. Primarily, object detection is generated on online maps during driving, while object classification is conducted using a trained classification model on an offline database. Most online maps are organized into grid maps with occupancy probabilities. Object-of-interest regions are usually represented as feature vectors and can be classified using classification models, such as support vector machine (SVM) [4] and AdaBoost [5], which are trained on offline databases.

Some methods are available that separately detect moving objects from stationary objects on grid maps using 3D point clouds of Light Detection And Ranging (LiDAR) sensors. They use the connectivity of occupancy probabilities or depth values to detect object regions [6,7]. Detecting object regions from charge-coupled device (CCD) images is conducted using object-region proposal generators. However, some problems are encountered. The first problem is related to missing proposals. If some object regions are not detected during the proposal-generation stage because of occlusion, viewing-point variations, weather issues or overlapping, the objects are very difficult to detect. The second problem pertains to efficiency of the segmentation results. Because previous methods only consider low or mid-level cues, they cannot detect desirable object proposals in near-object level regions.

To improve the detection and classification performance in intelligent vehicle systems, one possible approach is to fuse the measurements from several sensors. Managing incomplete measurements from different sensor modalities is necessary for accurate detection. The data-fusion scheme is generally categorized into two types, namely early and late fusion. The early-fusion method fuses two or more data by combining raw data or concatenating feature descriptors. Although the early-fusion method works well, it often cannot handle incomplete measurements. If one sensor modality becomes useless due to malfunctions, breakdown or severe weather conditions (e.g., a CCD sensor may lose confidence on rainy days), its measurements will be rendered ambiguous. The late-fusion method independently performs detection and classification from each sensor modality. Subsequently, the classified outputs are fused at the decision level for final classification. By using the decision-level fusion scheme for the object detection and classification task, we can prevent the autonomous driving system from becoming non-functional when information conflicts are introduced to more than one sensor. In addition, the reliability and plausibility of each sensor can be considered.

In this paper, we propose a new object-detection and classification method for a multi-layer LiDAR and a CCD sensor. The contributions of this work are two-fold: (1) an effective object-region proposal generation method; and (2) a decision-level fusion method for accurate object classification. For effective generation of the object-region proposals, we develop a new method to generate a small number of meaningful object-region proposals from the LiDAR and CCD sensor data. For the 3D point cloud data from the LiDAR sensor, supervoxel segmentation and region-growing methods are used [6], whereas color-flattening image segmentation and semantic grouping methods are proposed for the CCD sensor data. Semantic grouping is a process in which tiny partitions extracted from segment generation agglomerate with one another to form meaningful object regions. Our proposed color flattening is based on $L_{1}$ norm color transform [8]. Semantic grouping is performed using our own dissimilarity cost function between the color-flattened and original images.

**Figure 1:**
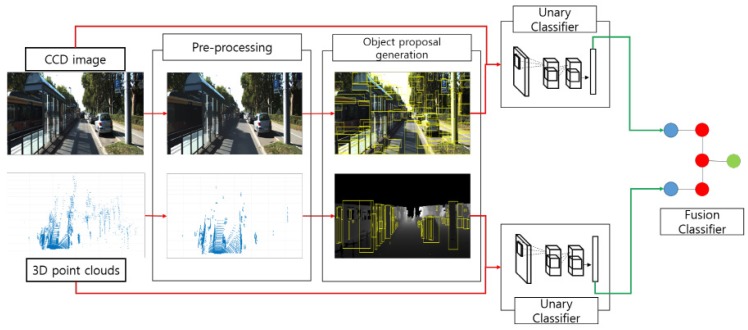
Overview of our work. Red arrows denote the processing of unary classifier for each sensor, and green arrows denote the fusion processing.

Figure 1 shows the overview of proposed method. For accurate object classification, we combine the results from the unary classifiers of each sensor at the decision level using a convolutional neural network (CNN). The main objective of the unary classifiers is to accurately recognize the class of object proposals on each sensor modality. Previous models of object category classification that used CNNs fed a fixed number of output layers into the final loss layer in their task. For example, all-passed output, which means the input is passed through all layers of networks, is widely used for feeding into the loss layer. For this output, however, little information loss might occur through the passing of several pooling layers. In contrast, the proposed CNN model, similar to unary classifiers, generates a convolutional cube from more than one convolutional layer of a pre-trained CNN model as image representations. From the extracted object proposals obtained from the proposal generations, ROI pooling is applied on the convolutional cube to feed them into a fine-tuned classification network comprising two convolutional layers, two fully-connected layers and a softmax layer. Subsequently, to fuse the two detection and classification results of the LiDAR and CCD sensors, we feed the final softmax result vectors and their convolutional cube into the fusion CNN. By fusing the multi-sensor modalities, the detection and classification failures can be compensated. In addition, fusing the multi-sensor in the decision level makes it more stable when information conflict occurs in each modality when compared to feature-level fusion schemes.

The remainder of this paper is organized as follows. Section 2 presents the related works. Section 3 provides an overview of our work. Section 4 and Section 5 describe the details of the pre-processing and object-region proposal-generation methods on each sensor. Our decision-level fusion method is presented in Section 6. Section 7 discusses the experimental result of the proposed work on a KITTI benchmark dataset [9]. Finally, the conclusion and future works are presented in Section 8.

## 2. Related Work

Object-proposal generation: The object detection and classification tasks can be divided into object-region proposal generation and proposal region classification. To extract the object-region proposals, one possible approach is to use the sliding-window method. The sliding window has been used in a wide range of detection tasks for faces [5,10,11,12], pedestrians [13,14,15,16,17] and cars [18,19]. Although the sliding window can search whole image regions (i.e., the recall rate is 100%), it generates a very large number of proposals (e.g., approximately 100,000 from a 640×480 image).

To reduce the number of object-region proposals, new approaches have been proposed, namely objectness [20], category-independent object proposals (CIOP) [21], constrained parametric min-cuts (CPMC) [22], selective search [23], EdgeBox [24], BInarized Normed Gradients (BING) [25] and multi-scale combinatorial grouping (MCG) [26]. The objectness method [20] measures the objectness score to distinguish whether a window region belongs to a background or an object. The category-independent method [21] extracts object regions using the graph-cut segmentation method and then ranks them to select a well-represented object-region proposal among the overlapped proposals. The CPMC method [22] ranks the plausibility of each segment to determine whether the foreground segments follow good object hypotheses or not. The selective search [23] hierarchically segments an image using the color, texture and size of each segment. EdgeBox [24] extracts object proposals using edge segmentations. In addition, it focuses on object boundaries for object-level proposals. The multi-scale combinatorial grouping method [26] segments an image in the hierarchical scale pyramids, and all of the segmentation results are then applied into the combinatorial grouping. Some previous works proposed the extraction of moving objects from video sequences [27,28,29,30] for traffic scenes. Because the amount of measured data in intelligent vehicle environments is larger than that in other applications, we need to design a new method to extract a smaller number of proposals than the previous methods.

Object detection and classification: Recently, object detection and classification methods that use CNN architecture have been thoroughly researched. AlexNet [31] won the ImageNet classification competition using CNN. Further, various works have achieved high classification performance on large-scale datasets [32,33,34], such as the ImageNet Large Scale Visual Recognition Challenge (ILSVRC) and PASCAL Visual Object Classed (VOC). Some previous works focused on their performance, while some architecture designs are available in the literature that focused on reducing the computational costs. “You only look once” (YOLO) [35] and faster R-CNN [34] constructed a CNN architecture that simultaneously performs object proposal generations and class classifications to reduce the computational times. Huang and Chen [28,29] proposed a variable-bandwidth network and a probabilistic neural network for traffic monitoring systems. However, if a model focuses on execution times rather than performance, the classification performance may be affected.

Detection on multi-sensor modality: In particular, for the scene-level detection and classification tasks, various sensor modalities are used. The RGB-depth sensor is widely used for indoor scene recognition [36,37,38], whereas LiDAR-stereo vision [39,40], LiDAR-CCD [41], LiDAR-radar [42] and LiDAR-radar-stereo vision [43] are used for outdoor scenes. For accurate classification, two or more input measurements are combined. The fusion methods are divided into two categories, namely early and late fusion. In the early fusion method, the measurements are fused by mapping them together, or by concatenation, or probabilistic fusion [41,44,45]. However, the early fusion method suffers from problems of non-overlapping regions and uncertainties. To solve these problems, the decision-level fusion method is used as a late fusion method. Chavez-Garcia and Aycard [46] proposed an evidential framework to improve the detection and tracking of moving objects by managing the uncertainty. Cho et al. [43] independently extracted data features using target information from sensors and combined the entire target information for movement classification and tracking of moving objects. The transferable belief model was used to combine the sensor measurements by managing the uncertainty [47]. In the present study, we use the CNN framework to jointly consider the classification performance of each sensor modality, as well as the uncertainties.

## 3. Overview

In this section, we describe the algorithm used in our proposed method, as shown in Figure 1. The inputs of our method consist of a CCD sensor and 3D point clouds from a multi-layer LiDAR, which are taken from a KITTI benchmark dataset [9]. The KITTI benchmark dataset also provides synchronized and calibrated data. Table 1 lists the notations used in our method. Our method consists of three phases: (1) pre-processing; (2) object-region proposal generation; and (3) classification of the object-region proposals.

Pre-processing: For the CCD image input, color flattening is performed, which makes the image assume a monotonous color and is useful in obtaining desirable segmentation results.

For the point-cloud input, we transform the 3D point clouds into 3D occupancy voxel spaces. This transformation reduces the noise in the point clouds, i.e., only the obviously reflected point measurements are acquired.

Object-region proposal generation: We perform segmentation of the color-flattened image. However, the initial segmentation results are not satisfactory with respect to the detection of meaningful objects. Therefore, we perform semantic grouping using a dissimilarity cost function from the pixel values of both the color-flattened and original images. These results are the object-region proposals from the CCD sensor data.

In the 3D occupancy voxel space, we extract the supervoxels using the voxel cloud connectivity segmentation (VCCS) [6]. VCCS uses a gradient-seeding methodology to segment point clouds. The resulting supervoxels are fine-level segments with a fixed size. Subsequently, we perform region growing on the supervoxels to obtain object-level segments using the occupancy connectivity because the supervoxels do not express meaningful cues. These results are the object-region proposals from the 3D point clouds.

Classifying object proposals: To classify the object proposals, we fuse the classification results from the unary classifiers of the LiDAR and CCD sensors using CNN. The unary classifiers are modeled using CNN models with the same network architecture. The proposed CNN models are generated with two phases consisting of image representation and classification networks. First, to represent the input data, we extract a convolutional cube that has more than one convolutional layer of pre-trained CNN models. From the convolutional cube of the input data, object regions from the object-proposal generations are extracted using ROI pooling. Then, the convolutional cube extracted from the proposal regions is fed into a classification network that includes two convolutional layers, two fully-connected layers and a softmax layer. To fuse the results from the two separate unary classifiers, we propose a CNN model that uses the convolutional cube and softmax results of the sensor modalities as input.

## 4. Pre-Processing

In this section, we present two pre-processing schemes of the CCD image and 3D point cloud data, such as the $L_{1}$ norm-based color flattening and 3D occupancy voxel spaces.

### 4.1. $L_{1}$ Norm-Based Color Flattening

Before applying the segmentation, we generate a color-flattened image from CCD sensor image I. Our color flattening is based on the $L_{1}$ image transform. However, the color flattening based on the $L_{1}$ image-transform method proposed by Bi et al. [8] was very costly. Therefore, we propose a modified color-flattening $L_{1}$ image transform by defining an energy function as follows:

(1) $$E\left(f\right)=E_{d}+E_{p},$$

where $E_{d}$ is the data term that considers the pixel-wise intrinsic similarity. $E_{p}$ refers to the local smoothness as the pairwise term.

The pixel-wise intrinsic similarity in the color-flattened image can avoid a negative solution where entire pixels are flattened with the same color values. To do this, the data term measures the similarity between the transformed and original images, which should be a minimum. We let $z^{*}$ and *z* represent a concatenated vector of all pixel values in transformed image $I_{c}$ and original image *I*, respectively. Data term $E_{d}$, which measures the pixel-wise intrinsic similarity, is defined as follows:

(2) $$E_{d}={\left|\right|z^{*}-z\left|\right|}_{2}^{2}.$$

The pairwise term, which measures the local smoothness, totals the weighted $L_{1}$ differences between adjacent pixel pairs. The $L_{1}$ difference between the pixel pairs of transformed image $I_{c}$ should be small for desirable flattening generation, i.e., neighboring pixels that have similar colors from original image I are assigned larger weights.

Let $x_{i}$ be the RGB vector at pixel position $p_{i}$ of transformed image $I_{c}$. If we consider a neighboring system, such as a set of m×m adjacent pixels of $p_{i}$, the pairwise term between $x_{i}$ and $x_{j}$ is defined as follows:

(3) $$E_{p}=\sum_{i=1}^{n}\sum_{j=1}^{(m\times m)-1}\omega_{i,j}{\left|\right|x_{i}-x_{j}\left|\right|}_{1},$$

where *n* is the number of pixels in image $I_{c}$. Weight $\omega_{i,j}$ is defined as follows:

(4) $$\omega_{i,j}=\exp\left(-\frac{{\left|\right|f_{i}-f_{j}\left|\right|}_{2}^{2}}{2\sigma^{2}}\right),$$

Where $f_{i}={\left[\kappa \times l_{i},a_{i},b_{i}\right]}^{T}$ from the CIELab color space of $p_{i}$. *κ* is a constant, which is related to the luminance variations. Using this constant, we can complexly suppress the luminance variations. If κ<1, the pairwise energy term becomes less sensitive to luminance variations. In our work, we set *κ* and *σ* to 0.3 and 1.0, respectively. To set each parameter, we iteratively run the proposed method using different parameter values on the KITTI object validation dataset. Based on the results of the operation using different parameter settings, we set *κ* and *σ* to 0.3 and 1.0, respectively. Subsequently, we validate the parameter settings using another validation set to verify that no overfitting occurs in the first validation dataset.

Let $M=\{M_{ij}\}$ be an $m_{l}\times n$ matrix. If $p_{j}$ is located in neighboring pairs m×m of $p_{i}$, $M_{ki}=\omega_{i,j}$ and $M_{kj}=-\omega_{i,j}$. To optimize the weights in Equations (3) and (4), we use the linear form of the least squares as follows:

(5) $$\begin{array}{c} z^{k+1}=arg\min_{z}\left(\beta E_{d}+\lambda_{p}{\left|\right|d_{k}-Lz-b_{k}\left|\right|}_{2}^{2}\right),\\ L=\left[\begin{array}{ccc} M & & \\ & M & \\ & & M \end{array}\right], \end{array}$$

where $d_{k}$ and $b_{k}$ are intermediate variables introduced by the split Bregman method [48]. To solve Equation (5), a normal sparse linear system is used. $\lambda_{p}$ controls the weight of the $L_{1}$ energy terms of the least square form. Algorithm 1 shows the steps to minimize Equation (5).

**Algorithm 1** Split Bregman for color-flattening.1:**procedure** COLORFLATTEN(*ϵ*,$\lambda_{p}$)2:    **Initial:** $z^{0}=z^{in}$; $d_{0},b_{0}=0$;3:    **while** $\left|\right|z^{k}-z^{k-1}{\left|\right|}_{2}^{2}>\epsilon$ **do**
4:        $A=\beta I_{3n\times 3n}+\lambda_{p}L^{T}L$
5:        $v=\beta z^{in}+\lambda_{p}L^{T}\left(d_{k}-b_{k}\right)$  6:        **Update** $z^{k+1}$ by solving $Az^{k+1}=v$  7:        $d_{k+1}=$**Shrink**($Lz^{k+1}+b_{k},\frac{1}{\lambda_{p}}$)  8:        $b_{k+1}=b_{k}+Lz^{k+1}-d_{k+1}$  9:        k=k+1  10:    **end while** 11:    **return** $z^{k}$  12:**end procedure** 13:**procedure** SHRINK(y,γ)  14:    **return** $\frac{y}{\left||y|\right|}\times \max(||y||-\gamma ,0)$  15:**end procedure**


### 4.2. The 3D Occupancy Voxel Spaces

As raw data, the 3D point clouds can have many noisy reflectance particles. If the given point clouds contain significant noise, erroneous object partitions will be generated in the segmentation task, i.e., the number of meaningless partitions will increase. This incurs additional computational costs to achieve desirable segmented results. To reduce the noise, 3D point clouds with adjacent positions are transformed to discrete 3D occupancy voxel spaces. Let $\gamma_{i}$ denote the *i*-th 3D point data of the 3D point clouds, which is composed of $\left[x_{i},y_{i},z_{i}\right]$. The *i*-th voxel $\Gamma_{i}$ includes the reflected particles with an $m_{v}^{x}\times m_{v}^{y}\times m_{v}^{z}$ size. If the voxel size is too small, noise reduction may be difficult. On the other hand, if the voxel size is too large, the meaningful shape of the object may be suppressed, i.e., segmentation will not be adequately performed. In our work, we set $m_{v}^{x}=m_{v}^{y}=m_{v}^{z}=0.1$ m.

The occupancy probability of each voxel $p(\Gamma_{i})$ can be measured as follows:

(6) $$p\left(\Gamma_{i\in N_{\Gamma }}\right)=\frac{\sum_{j=1}^{N}\gamma_{i,j}}{\Gamma_{i}^{\max}},\;\gamma_{i,j}\in \Gamma_{i},$$

where $N_{\Gamma }$ is the number of voxels in a 3D point cloud and $\Gamma_{i}^{\max}$ denotes the possible number of reflectance particles in voxel $\Gamma_{i}$. According to the qualification of a LiDAR sensor, we can learn the number of reflectance particles that can be included in a voxel. $\gamma_{i,j}$ is the *j*-th particle of $\Gamma_{i}(\gamma_{i,j}=1$ when a *j*-th laser is reflected by obstacles; otherwise, $\gamma_{i,j}=0$).

## 5. Object-Region Proposal Generation

### 5.1. Object-Region Proposal from the CCD Sensor

To obtain the object-region proposals from an image, we first segment color-flattened image $I_{c}$. The segmented image has several very small partitions; therefore, each partition has only mid-level visual cues. Thus, we require a grouping task to obtain more semantic information from the segmented partitions. To achieve this, we design a simple dissimilarity function between adjacent partitions $s_{i}$ and $s_{j}$.

Let a segmented partition set of the color-flattened image be $S=\{s_{1},s_{2},\cdots ,s_{N_{s}}\}$, where $s_{i}$ denotes the *i*-th segmented partition and $N_{s}$ is the number of partitions. Each segmented partition has its set of spatially-connected neighborhood partitions $N(s_{i})$, where $\left(s_{i},s_{j}\right)\in N(s_{i})$ if $s_{i}$ and $s_{j}$ are adjacently located. At this point, the dissimilarity function is defined as follows:

(7) $$\psi_{i,j}=\alpha_{c}\psi_{i,j}^{c}+\alpha_{t}\psi_{i,j}^{t},$$

where $\psi_{i,j}^{c}$ and $\psi_{i,j}^{t}$ capture the color and texture dissimilarity between adjacent partitions, respectively. $\alpha =[\alpha_{c},\alpha_{t}]$ is the corresponding weight constant that will be learned. If $\psi_{i,j}<\theta_{d}$, adjacent partitions $s_{i}$ and $s_{j}$ will be grouped. $\theta_{d}$ denotes a threshold value that should be learned.

For the color dissimilarity, we construct a histogram with 25 bins from each partition. Then, we concatenate them into a 75-bin histogram $z^{c}$. At this point, we use the color value from mean image $I_{\mu }$ between original image *I* and color-flattened image $I_{c}$ as HSV color values because this will be useful in generating desirable grouping $I_{\mu }\left(x,y\right)=\beta_{c}\times I(x,y)+\left(1-\beta_{c}\right)\times I_{c}\left(x,y\right)$, where $\beta_{c}$ is a weight constant. We set $\beta_{c}=0.6$. The $L_{1}$ norm is used to measure the color dissimilarity: $\psi_{i,j}^{c}=\left|\right|z_{i}^{c}-z_{j}^{c}{\left|\right|}_{1}$.

The texture dissimilarity is measured by scale-invariant feature transform (SIFT) histogram $z^{t}$, which is constructed from Gaussian derivatives with eight orientations at σ=1 for each RGB color channel. In each orientation, the histogram is constructed with 10 bins. Consequently, the SIFT histogram has 240 bins. The textures of original image *I* are used for the texture dissimilarity measurement because the partitions of color-flattened image $I_{c}$ do not include any textures. Similar to the color dissimilarity measurement, we use the $L_{1}$ norm to compute the texture dissimilarity: $\psi_{i,j}^{t}=\left|\right|z_{i}^{t}-z_{j}^{t}{\left|\right|}_{1}$.

To optimize the weights of the dissimilarity function (i.e., Equation (7)), we use the PASCAL VOC 2012 segmentation dataset [49]. In the given original image *I*, *S* and $S^{\prime }$ denote the ground truth of the segmented and inferred segmentation images obtained from the proposed method, respectively. We aim to find the optimized weight combination of $\alpha =[\alpha_{c},\alpha_{t}]$, which can optimally group the mid-level segment partitions into object-level segment partitions. From given $N_{s}$ training images, the objective function is defined as follows:

(8) $$\begin{array}{c} arg\min_{\alpha ,\xi_{n}\geq 0}\alpha^{T}\alpha +\lambda \sum_{n=1}^{N_{s}}\xi_{n}\\ s.t.\;\forall n\in \left[1,N_{s}\right],\;\forall S_{n}^{\prime }\\ \;\psi \left(I_{n},S_{n}^{\prime }\right)-\psi \left(I_{n},S_{n}\right)\geq \Delta \left(S_{n},S_{n}^{\prime }\right)-\xi_{n}, \end{array}$$

where Δ(·,·) denotes the structural loss between the ground truth of the segmented partition and the inferred segmented partition. λ≥θ is the regularization parameter predefined by a linear SVM, and $\xi_{n}$ is a slack variable.

**Figure 2:**
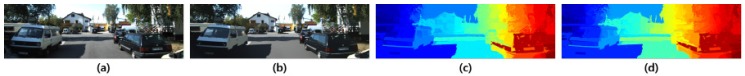
Procedure from pre-processing to semantic grouping on CCD image data. (**a**) Input image data; (**b**) color-flattened image; (**c**) segmented image using the graph-segmentation method; (**d**) semantic grouping using the dissimilarity cost function.

Finally, the set of object-region proposals of the image data can be acquired using the grouped partition set. Figure 2 shows our semantic grouping results.

### 5.2. Object-Region Proposal from the LiDAR Sensor

In the 3D point cloud data, VCCS [6] is first applied to occupancy voxel space Γ to generate fine-level segments. The supervoxels partitioned by the VCCS adhere to the object boundaries adequately. The VCCS uses a seeding methodology in 3D data spaces. In addition, by using color and geometric features, a flow-constrained local iterative clustering is applied to generate supervoxel spaces. For the decision-level fusion, we need to obtain the probability of each supervoxel instead of the color values of the original VCCS method. K-means clustering is used to generate the VCCS with two constraints. The first constraint is that the partitioned supervoxels are distributed according to the geometry information. The next one is that the supervoxels cannot be overlapped between more than two regions in the 3D space. Using the seed number selection, we determine the number of occupancy voxels.

**Figure 3:**
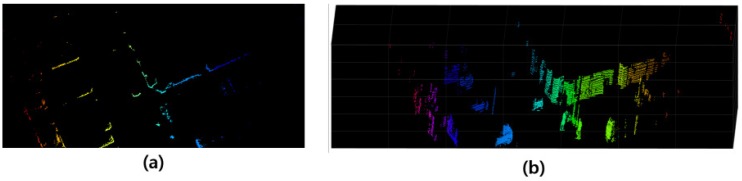
Segment generation on 3D point clouds. (**a**) 2D occupancy grid mapping results and (**b**) segmentation result on 3D point clouds.

The supervoxel regions extracted from the aforementioned method do not contain any meaningful information. To solve this problem, the supervoxels are first projected onto an [X,Z] 2D space with a grid size of 0.1 m × 0.1 m. Subsequently, the supervoxels are grouped. To connect the partitions as object-level segments, the difference in height (*Y*-axis value) of the supervoxels in each grid cell is computed. If the difference in height of a grid cell is above 0.1 m, the supervoxels of the grid cell are connected. Figure 3 shows an example of the segmentation generation in the 3D point clouds. From the given 3D origins, i.e., the height, width and length of each partition, we can obtain the object-region proposals of the 3D bounding boxes.

## 6. Classifying Object-Region Proposals

From the extracted bounding box sets of each sensor modality in Section 5, the object region proposals are classified using the unary classifiers. The unary classifiers are separately used for each sensor; however, they have the same network architecture. Subsequently, we fuse them using another CNN model by measuring the associations among the bounding boxes.

Directly training 3D point clouds using CNN is difficult because the 3D point clouds do not contain suitable information to classify the object classes from the object proposals. Further, the sparse point clouds contain ambiguously distributed shapes of the points in order to enable the classes of the object regions to be distinguished. To solve these issues, we project the 3D point clouds into a dense depth map using the up-sampling method [7].

### 6.1. Unary Classifier

**Figure 4:**
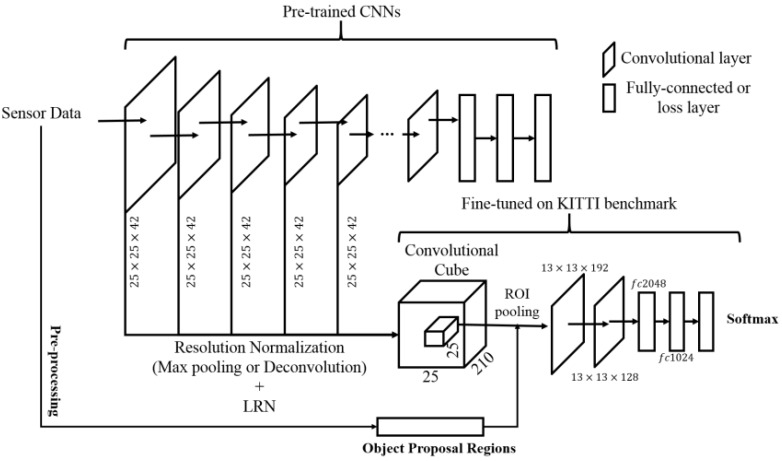
Proposed network architecture as unary classifiers.

CNN models have been successfully applied in a wide range of tasks. One of the benefits of the CNNs is the simultaneous direct learning of representation and estimation. We propose a CNN architecture for accurate classification of the object proposals. Figure 4 shows the architecture of the unary classifier used to classify the object proposals presented in Section 5.

The objects of the driving scenes contain large variations in their scales. Scale variations can be introduced by distances from the ego-vehicle and/or inter (or intra) types of objects in driving scenes. However, previous CNN models for detecting and classifying objects used the fixed output from the final layers of a model as a data representation. At this point, the fine features can be gradually ignored according to a passing layer, which includes some types of operations, such as convolution and pooling. In particular, if a small bounding box has passed entire layers, feature losses may be introduced owing to its low resolution. Therefore, to use the features of small objects, as well as objects with moderate sizes, we construct a convolutional cube from each input data as a data representation. The convolutional cube represents more than one convolutional output of some stacked convolutional layers, which is similar to HyperFeature [33]. Because the sizes of the outputs from each convolutional layer vary, we should individually sample them by applying different sampling layers to the stack outputs of the convolutional layers. The subsampling for the outputs of the convolutional layers with sizes larger than that of the convolutional cube is generated by max pooling layers. Meanwhile, a deconvolutional layer is used to up-sample the outputs of convolutional layers that are smaller than the size of the convolutional cube. Subsequently, local response normalization is used to normalize the entire output. Consequently, we can obtain the convolutional cube of the input data with a uniform scale.

The region of the object proposal presented in Section 5 is extracted from the convolutional cube of the input data using ROI pooling. Then, the convolutional cube of each object proposal is fed into a small CNN to classify their object category. This CNN is fine-tuned on a KITTI dataset. The network comprises two convolutional layers with max pooling layers, two fully-connected layers and one softmax layer.

### 6.2. Fusion Classifier

Bounding box association: To fuse the classification results extracted from each sensor datum, we need to find an association between the object bounding boxes. In this paper, we represent the association as basic belief assignment (BBA) [50,51]. The fusion of the classification results provided by each unary classifier leads us to benefit from the reliability by reducing the uncertainty.

We let $\alpha_{i,i\in n}$ and $\beta_{j,j\in m}$ denote the sets of the classification results of each bounding box provided by the image and 3D point cloud unary classifiers, respectively. The numbers of classified bounding boxes from CCD and LiDAR are *n* and *m*, respectively. The association is represented as an n×m matrix, namely the association matrix. Each cell of the matrix is the association component $m_{\alpha_{i},\beta_{j}}$ between the classification results of $\alpha_{i}$ and $\beta_{j}$, where i≤n;j≤m. The frame of discernment $\Omega_{C}=\left\{1,0\right\}$ of the classification results is used to represent the association and is defined as follows:

(9) $$\Omega_{C}=\left\{\begin{array}{cc} 1, & \mathrm{if}\;a_{i}\;\mathrm{and}\;b_{j}\;\text{are of the same class}\\ 0, & \mathrm{if}\;a_{i}\;\mathrm{and}\;b_{j}\;\text{are not of the same class}\\ \Omega , & \text{ignorance} \end{array}\right..$$

Therefore, $m_{\alpha_{i},\beta_{j}}\left(\left\{1\right\}\right)$ and $m_{\alpha_{i},\beta_{j}}\left(\left\{0\right\}\right)$ denote the association probability $P(\alpha_{i},\beta_{j})=1$ and $P(\alpha_{i},\beta_{j})=0$, respectively. The probability of association can be measured by the distance and class dissimilarity between $\alpha_{i}$ and $\beta_{j}$.

To measure distance $m_{\alpha_{i},\beta_{j}}^{d}$ between two classification results, we use the Mahalanobis distance because it includes the correlation of the distance set. The BBA of $m_{\alpha_{i},\beta_{j}}^{d}$ is measured as follows:

(10) $$m_{\alpha_{i},\beta_{j}}^{d}\left(\left\{1\right\}\right)=\nu \times d_{\alpha_{i},\beta_{j}},$$

(11) $$m_{\alpha_{i},\beta_{j}}^{d}\left(\left\{0\right\}\right)=\nu \times \left(1-d_{\alpha_{i},\beta_{j}}\right),$$

(12) $$m_{\alpha_{i},\beta_{j}}^{d}\left(\left\{\Omega \right\}\right)=1-\nu ,$$

where ν∈[0,1] denotes the evidence discounting factor and $d_{\alpha_{i},\beta_{j}}\in \left[0,1\right]$ is the Mahalanobis distance between $\alpha_{i}$ and $\beta_{j}$. To return to the larger value when the distance becomes smaller, we use the following function:

(13) $$d_{\alpha_{i},\beta_{j}}^{\prime }=e^{-\theta d_{\alpha_{i},\beta_{j}}},$$

where *θ* is a threshold factor that determines whether the distance is near or far.

For the class association between $\alpha_{i}$ and $\beta_{j}$, we use class dissimilarity. Although two bounding boxes $\alpha_{i}$ and $\beta_{j}$ have the same object class, they may include different objects. On the other hand, if $\alpha_{i}$ and $\beta_{j}$ belong to different classes, they are clearly different from each other. Therefore, we compute the dissimilarity as class association $m_{\alpha_{i},\beta_{j}}^{c}$. The frame of discernment for class association is the set Ω = {cars, pedestrians, cyclists}. The classification results of each unary classifier, which consist of the probability proportions about classes of interest, are transformed into a BBA mass function form using pignistic transformation [51], i.e., $m_{S}^{c}\in \left\{m_{S}^{c}\left(cars\right),m_{S}^{c}\left(pedestrians\right),m_{S}^{c}\left(cyclists\right)\right\}$, where $m_{S_{k}}^{c}\left(\cdot \right)$ denotes the mass of the class of the *k*-th bounding box obtained from the sensor modality *S*. The class dissimilarity is computed as follows:

(14) $$m_{\alpha_{i},\beta_{j}}^{c}\left(\left\{1\right\}\right)=0,$$

(15) $$m_{\alpha_{i},\beta_{j}}^{c}\left(\left\{0\right\}\right)=\sum_{A\cap B=\emptyset }m_{\alpha_{i}}^{c}\left(A\right)m_{\beta_{j}}^{c}\left(B\right),\forall A,B\in \Omega ,$$

(16) $$m_{\alpha_{i},\beta_{j}}^{c}\left(\left\{\Omega \right\}\right)=1-m_{\alpha_{i},\beta_{j}}^{c}\left(\left\{0\right\}\right).$$

Finally, we can obtain bounding box association $m_{\alpha_{i},\beta_{j}}$ using Yager’s combination rule [52], which is expressed as follows:

(17) $$\begin{array}{cc} m_{\alpha_{i},\beta_{j}}\left(\left\{\Omega_{C}\right\}\right) & =m_{\alpha_{i},\beta_{j}}^{\prime }\left(\left\{\Omega_{C}\right\}\right)+\kappa_{\alpha_{i},\beta_{j}}^{a},\\ m_{\alpha_{i},\beta_{j}}^{\prime }\left(A\right) & =\sum_{D\cap C=A}m_{\alpha_{i},\beta_{j}}^{d}\left(D\right)m_{\alpha_{i},\beta_{j}}^{c}\left(C\right),\\ \kappa_{\alpha_{i},\beta_{j}}^{a} & =\sum_{D\cap C=\emptyset }m_{\alpha_{i},\beta_{j}}^{d}\left(D\right)m_{\alpha_{i},\beta_{j}}^{c}\left(C\right), \end{array}$$

where *D* and *C* denote the bounding box spaces of each sensor. If two bounding boxes are set as the associated bounding box, the data of the bounding boxes are passed to our proposed fusion classifier.

**Figure 5:**
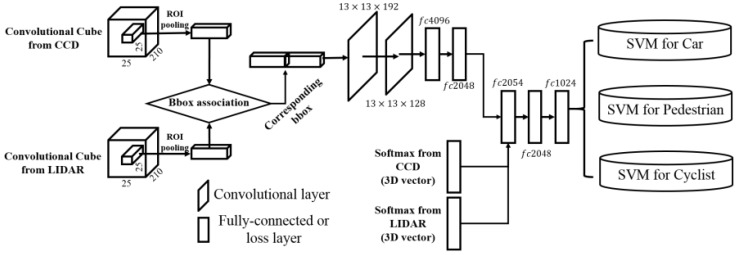
Architecture of the fusion network. Bbox denotes bounding box (Section 6.2).

Classifier for fusion results from unary classifiers: For the decision-level fusion, object-proposal generations and classifications are first independently run on each sensor modality. The next process is performed to fuse the decision results using a CNN model. Figure 5 shows the network architecture of the fusion classifier.

The fusion classifier takes two separate input columns that include convolutional cubes and category probabilities of the softmax layers from each sensor modality. The input of the first column is a convolutional cube in which the convolutional cubes from each sensor modality are concatenated. By passing two convolutional layers and two fully-connected layers, the concatenated convolutional cube becomes a 2048-dimensional vector. This vector is concatenated using two-class probability vectors. Subsequently, a 2054-dimensional vector (2048 + 3 + 3) is fed into two fully-connected layers and a binary class SVM for the final fusion classification.

## 7. Experimental Results

In this section, we first validate the design choices of the proposed method. In addition, we describe the comparative evaluations of the proposed method relative to the baseline algorithms of the KITTI benchmark dataset.

### 7.1. Setup

Dataset: To fine-tune the classification network phases of the unary classifiers and the fusion network, we used the training dataset of the KITTI benchmark. The employed categories comprised cars, pedestrians and cyclists. At this point, the cars category included the truck and bus categories of the KITTI dataset. The evaluations of the proposed method were conducted on 15% of the training dataset of the KITTI benchmark non-overlapped with 85% of the training dataset.

At the training time, we cropped the 3D object boxes from the 3D point clouds, and each object box was then mapped onto the 3D point clouds. Subsequently, the dense depth maps, employed as the training dataset, were trained using CNNs. During the test time, the pre-processing phase and the object-region proposal generation were performed in 3D spaces, whereas the dense depth maps were passed out to the classification model. The cropped object regions from the image dataset were simply used to train the CNNs for the image data.

Implementation details: We implemented the proposed model using MATLAB on an Intel-Core i5-4570 dual-core 3.20-GHz processor with 8.00 GB of RAM and an NVIDIA GeForce GTX 650 graphic card with 3.7 GB of memory for CUDA computations. The machine used to train the CNNs and conditional random fields (CRFs) is an Intel-Core i7-6700 quad-core 4.0-GHz processor with 64.00 GB of RAM and an NVIDIA GeForce Titan X graphic card with 12 GB of memory for CUDA computations. We trained the CNNs using Caffe [53], which is a widely-used deep-learning tool.

To extract the convolutional cubes from each sensor modality, we used the VGG16 [54] architecture. No pre-trained CNN models are available for the dense depth map from the 3D point cloud data, whereas the VGG16 has been pre-trained using ImageNet. Therefore, we extracted the convolutional cube of the 3D point cloud using the cross-feature extraction method [55]. Gupta et al. [55] have proposed a method for extracting data representation from another pre-trained CNN model trained on different data modalities.

To reduce localization errors, we trained a linear regression model for the bounding box fitting to the objects, which was employed in the deformable part model (DPM) [56].

### 7.2. Evaluation

We evaluated our proposed method to validate our various design choices. The proposal generation part presents the detection of the effectiveness of the presented proposal-generation methods. In the representation architecture discussion, we present our observation on which pre-trained CNNs could precisely represent the multi-class objects. To show the effectiveness of fusing multiple sensor modalities, we present our conducted process in the data modality part. Finally, we evaluate our proposed fusion schemes by comparing it with the usage of a single sensor modality or other fusion schemes, as presented in the fusion scheme paragraph. The basic configuration of our proposed method is listed in the row ours in Table 2.

Proposal generation: Table 3 lists the recall rates and the number of proposals. Our proposed method extracted approximately 1000 proposals from the image data and 65 proposals from the 3D point clouds. The passed-out object regions to the final fusion were approximately 500 bounding boxes obtained using bounding box association. Although the number of proposals was smaller than that of the other methods, we achieved approximately 90% recall for the cars class. We outperformed the other methods in each class by 90%, 88% and 86% for cars, pedestrians and cyclists, respectively, in the moderate level. These results may have been obtained from our semantic grouping scheme. We can sufficiently segment 3D point cloud and image data as object-level regions.

To evaluate the performance of our method according to the usages of the proposal generation methods, we compared our mean average precision (mAP) with the other proposal generation methods. The compared models were as follows: sliding window ($model_{1}$), CIOP ($model_{2}$), objectness ($model_{3}$), original selective search ($model_{4}$), CPMC ($model_{5}$), MCG ($model_{6}$) and EdgeBox ($model_{7}$). As shown in the ours and $model_{1,\cdots ,7}$ rows in Table 4, ours more accurately detected and classified the entire object classes than the other models. We conclude that our proposal-generation methods can precisely extract object regions at object levels.

Representation architecture: First, we compared the pre-trained network architecture to represent objects. For comparison, we used AlexNet, which was pre-trained on ImageNet Large Scale Visual Recognition Competition 2012 (ILSVRC2012) ($model_{8}$), because it is a widely-used network that includes smaller layers than VGG16. Similar to ours, we extracted five convolutional layers to construct ConvCube from the pre-trained AlexNet. The ours and $model_{8}$ rows in Table 4 show that the ConvCube extracted from the larger network (VGG16) can represent objects more precisely.

In addition, we compared the accuracy in constructing ConvCube according to the usages of convolutional layers. The comparison models were as follows: output of the output of first convolutional layer (conv1) only ($model_{9}$), conv5 only ($model_{10}$), the ouput of seventh fully connected layer (fc7) only ($model_{11}$) and conv5fc7 ($model_{12}$) layers from the pre-trained VGG16. To feed ConvCube into the classification network with uniform scales, we applied the sampling methods into each layers, such as those presented in Section 6.1. As shown in the ours and $model_{9,\cdots ,12}$ rows in Table 4, we can conclude that mAP is the highest when the outputs of the entire convolutional layers were used to construct ConvCube. This result demonstrates that information losses can be reduced by using all convolutional layers.

Data modality: In this experiment, we compared the accuracy differences generated from the sensor modality. $model_{13}$ and $model_{14}$ are generated on CCD and LiDAR, respectively. As shown in the ours and $model_{13,14}$ rows in Table 4, the model that fuses the classification results of the CCD and LiDAR modalities outperformed the unary classifiers of the CCD and LiDAR sensors by 4.09% and 15.86% mAP, respectively. We conclude that the detection and classification failures of each unary classifier can be compensated by fusing two sensor modalities at the decision level.

Fusion scheme: As target models for the decision-level fusion scheme, we used the transferable belief model (TBM) [47] ($model_{15}$) and conditional random fields (CRFs) [57] ($model_{16}$). For TBM, the results from each unary classifier were combined using the belief model. On the other hand, the results from each unary classifier and the final fully-connected layer were fed into the CRF model to consider joint probability. When the fusion of the unary classifiers was performed by TBM and CRF, we could observe that the final classification results were lower than the result of the CCD-only classifier. In addition, to compare the proposed method with the other classifiers that fuse features at an early decision level, we implemented 3D Object Proposal (3DOP) [58] ($model_{17}$). The 3DOP extracts object proposals with a higher recall rate using depth information from a stereo vision sensor. Therefore, we extract the depth information from the 3D point clouds.

The performance comparison is described as follows: as listed in the rows in Table 4, the mAPs of the entire object classes in the ours row are the highest when compared with the other models. Further, ours can classify object classes more accurately than $model_{17}$.

State-of-the-art comparisons: Table 5 lists the comparison results between the proposed method and state-of-the-art methods. We achieved improvement in average precision (AP) of 89.34%, 70.84% and 72.98% over the entire classes for the cars, pedestrians and cyclists, respectively, in the moderate level. As listed in Table 5, the results of the pedestrians and cyclists classes were slightly improved over those of the baseline methods. This result demonstrates that the 3D point clouds cannot completely measure obstacles located far from the sensors. Further, because the gap between scans of two laser beams is widely spread according to the distances, objects corresponding to pedestrians and cyclists could be missed.

**Figure 6:**
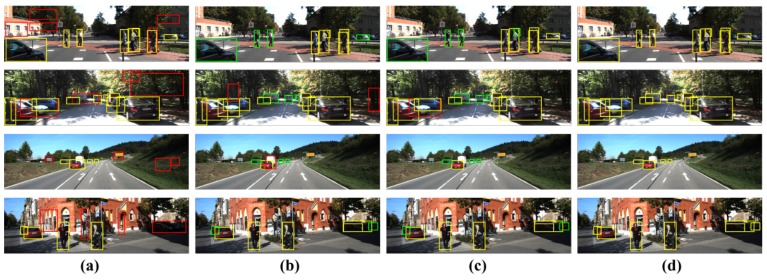
Qualitative results of our proposed method. We projected the classification results on the image data. (**a**) The results of CCD unary classifier. (**b**) The results of LiDAR unary classifier. (**c**) The results of $model_{17}$. (**d**) The results of proposed method. Each box indicates the following: yellow box: correctly-detected and -classified objects; red box: failures; green box: un-detected objects.

Qualitative results: Figure 6 shows the qualitative examples. Figure 6a,b shows the results of the unary classifiers generated on the CCD and LiDAR sensor, respectively. Figure 6c shows the qualitative results of $model_{17}$. Non-object regions are classified as objects of interest in the results on the unary classifier of the CCD sensor because a large number of object proposals are still generated. On the other hand, some objects are missing in Figure 6b because of the limitations of the sensor measurements of the LiDAR sensor. Figure 6c shows that the objects located in the regions with information conflicts are not detected and classified.

Figure 6d shows the results of the proposed method. In the proposed method, the final classification results are projected onto the input RGB image. The position of the 3D bounding boxes can be estimated using a calibration matrix between the 3D point clouds and the image. The feature-fusion scheme (Figure 6c) occasionally misses the objects located in the information conflict areas; however, the proposed method, which uses the decision-level fusion scheme, could more accurately detect and classify the objects regardless of the information conflicts.

## 8. Conclusions and Future Works

In this paper, we proposed a new object-region proposal-generation method for object detection and a decision-level fusion method for accurate classification of objects for multi-sensor modalities of intelligent vehicles. The pre-processing tasks, such as color flattening and 3D occupancy voxel space, were used to reduce noises. Then, we performed segmentation and grouping to generate a small number of object-region proposals. Thereafter, the object-region proposals were classified using independent unary classifiers. Finally, we fused the results of each unary classifier using a CNN model.

The experimental results on the KITTI benchmark dataset showed that more meaningful object-region proposals were extracts, while the number of proposals was reduced when compared with those of the previous methods. The performance of our object classification method fused from the CCD and LiDAR sensors was better than that of the other methods. The limitations of our proposed method are as follows: (1) it cannot be generated in real time due to the color flattening and proposal generation in 3D point clouds; (2) the classification accuracies on pedestrians and cyclists remain low due to the incomplete data from the LiDAR sensor modality.

Various previous methods focused on the improvement of detection performance. Although the proposed model requires a bit more computational cost, the computational cost could be reduced by implementing GPU-based approaches. Although the proposed method could reduce the failures generated on the feature-level fusion schemes and unary classifiers, the failures of each unary classifier slightly influence the final classification results. To address this problem, we will improve the accuracy of the proposed method in terms of the detection rate of pedestrians and cyclists classes by dealing with the confidence of the sensor information for effective classification.

## Figures and Tables

**Table 1:** The notation of the used parameters and functions.

Section	Parameters or Functions	Descriptions
	E(f)	Energy function to generate color-flattening, $E\left(f\right)=e_{d}+E_{p}$.
	$E_{d}$	Data term of energy function for pixel-wise intrinsic similarity.
	$z^{*}$	A concatenated vector of all pixel values in transformed image $I_{c}$.
	*z*	A concatenated vector of all pixel values in original image *I*.
4.1	$E_{p}$	Smoothness term of energy function.
	$x_{i}$	A 3-dimensional vector of the RGB values at pixel position $p_{i}$ of transformed image $I_{c}$.
	$\omega_{i,j}$	Weights to the difference between $x_{i}$ of pixel position $p_{i}$ and $x_{j}$ of the neighboring pixel $p_{j}$ of $p_{i}$.
	$f_{i}$	A 3-dimensional vector of the CIELab color space of $p_{i}$.
	*κ*	A constant related to the luminance variations.
	*M*	The $m_{l}\times n$ matrix consists of $\omega_{i,j}$ and $-\omega_{i,j}$.
	$d_{k}$ and $b_{k}$	Intermediate variables of the split Bregman method.
	$\gamma_{i}$	$\gamma_{i}=\left[x_{i},y_{i},z_{i}\right]$, the *i*-th 3D point data of 3D point clouds.
4.2	$\Gamma_{i}$	The *i*-th voxel includes the reflected particles with a size of $m_{v}^{x}\times m_{v}^{y}\times m_{v}^{z}$.
	$N_{\Gamma }$	The number of voxels in a 3D point cloud.
	$\Gamma_{i}^{\max}$	The possible number of reflectance particles in voxel $\Gamma_{i}$.
	S	A set of segmented partition of the color-flatted image.
	$N_{s}$	The number of segmented partitions.
	$N(s_{i})$	Set of spatially-connected neighborhood partitions of the $s_{i}$ partition.
	$\psi_{i,j}$	The dissimilarity function to group the adjacent partitions.
	$\psi_{i,j}^{c}$	$\psi_{i,j}^{c}=\left|\right|z_{i}^{c}-z_{j}^{c}{\left|\right|}_{1}$; the color dissimilarity between the adjacent partitions.
	$\alpha_{c}$	A weight constant for the color dissimilarity.
5.1	$z^{c}$	75-bin color histogram measured from the mean image $I_{\mu }$.
	$\psi_{i,j}^{t}$	The texture dissimilarity between the adjacent partitions.
	$\alpha_{t}$	A weight constant for the texture dissimilarity.
	$z^{t}$	240-bin SIFT histogram of original image *I*.
	$\theta_{d}$	A threshold value for grouping adjacent partitions.
	*S* and $S^{\prime }$	The ground truth of the segmented and inferred segmentation images from the proposed method.
	$N_{s}$	The number of training images to find $\alpha =[\alpha_{c},\alpha_{t}]$.
	Δ(·,·)	The structural loss between the ground truth and the inferred segmented partition.
	$\alpha_{i,i\in n}$	The classification results of each bounding box provided from the image.
6.2	$\beta_{j,j\in m}$	The classification results of each bounding box provided from the 3D point clouds.
	$m_{\alpha_{i},\beta_{j}}$	The association component between $\alpha_{i}$ and $\beta_{j}$.

**Table 2:** Comparison models used to evaluate the proposed method. ConvCube, convolutional cube; CIOP, category independent object proposals; CPMC, constrained parametric min-cuts; MCG, multiscale combinatorial grouping; TBM, transferable belief model; CRF, conditional random field; 3DOP, 3D object proposal.

Model	Proposal Generator	Representation	Representation Usage	Modality	Fusion Scheme
$model_{1}$	Sliding Window	VGG16	ConvCube	CCD + LiDAR	CNN
$model_{2}$	CIOP	VGG16	ConvCube	CCD + LiDAR	CNN
$model_{3}$	Objectness	VGG16	ConvCube	CCD + LiDAR	CNN
$model_{4}$	Selective Search	VGG16	ConvCube	CCD + LiDAR	CNN
$model_{5}$	CPMC	VGG16	ConvCube	CCD + LiDAR	CNN
$model_{6}$	MCG	VGG16	ConvCube	CCD + LiDAR	CNN
$model_{7}$	EdgeBox	VGG16	ConvCube	CCD + LiDAR	CNN
$model_{8}$	Proposed Generator	AlexNet	ConvCube	CCD + LiDAR	CNN
$model_{9}$	Proposed Generator	VGG16	conv1	CCD + LiDAR	CNN
$model_{10}$	Proposed Generator	VGG16	conv5	CCD + LiDAR	CNN
$model_{11}$	Proposed Generator	VGG16	fc7	CCD + LiDAR	CNN
$model_{12}$	Proposed Generator	VGG16	conv5 + fc7	CCD + LiDAR	CNN
$model_{13}$	Proposed Generator	VGG16	ConvCube	CCD	×
$model_{14}$	Proposed Generator	VGG16	ConvCube	LiDAR	×
$model_{15}$	Proposed Generator	VGG16	ConvCube	CCD + LiDAR	Decision-TBM
$model_{16}$	Proposed Generator	VGG16	ConvCube	CCD + LiDAR	Decision-CRF
$model_{17}$	3DOP	3DOP	3DOP	CCD + LiDAR	Feature-3DOP
ours	Proposed Generator	VGG16	ConvCube	CCD + LiDAR	CNN

**Table 3:** Recall of each object-region proposal method. # of Bbox denotes the number of bounding box in the KITTI data. The bold numbers in the recall column represent the highest recall except for the sliding window (because the sliding window always contains 100%). The bold component in # of Bbox represent the smallest number among the entire methods.

Method	Recall (%)			# of Bbox
	Cars	Pedestrians	Cyclists	
Sliding window	100	100	100	$10^{8}\times n$
CIOP	64.4	59.8	59.9	$10^{3}\times n$
${\mathrm{objectness}}_{1000}$	66.9	60.4	60.1	$10^{2}\times n$
Selective search	70.4	66.8	68.7	$10^{6}\times n$
CPMC	71.7	67.4	68.6	$10^{3}\times n$
MCG	76.6	78.9	74.8	$10^{4}\times n$
EdgeBox	85.2	84.3	82.5	$10^{4}\times n$
Ours (CCD)	88.4	85.4	84.8	$10^{3}\times n$
Ours (LiDAR)	71.8	63.3	64.2	**70**
Ours	**90.8**	**88.7**	**86.5**	500

**Table 4:** Comparison of the proposed method with design-varied models. The best scores are boldfaced.

Model	Cars			Pedestrians			Cyclists		
	Easy	Moderate	Hard	Easy	Moderate	Hard	Easy	Moderate	Hard
$model_{1}$	90.98	88.64	79.88	82.84	69.55	66.42	82.12	71.48	64.55
$model_{2}$	90.7	83.67	79.78	80.54	68.07	65.23	80.86	68.59	63.54
$model_{3}$	91.34	85.28	77.42	81.71	68.54	61.19	78.21	68.77	63.77
$model_{4}$	85.88	87.74	79.01	79.59	68.45	62.66	82.65	65.12	61.38
$model_{5}$	91.39	87.78	75.7	75.25	66.35	61.27	76.24	66.93	63.39
$model_{6}$	89.42	82.94	77.1	80.94	67.93	61.58	79.07	66.67	63.27
$model_{7}$	85.68	87.82	79.57	81.53	65.02	65.94	78.67	67.89	60.81
$model_{8}$	87.43	84.44	75.42	73.2	65.28	64.55	77.51	66.74	60.15
$model_{9}$	86.29	81.26	73.52	72.86	63.04	60.31	74.69	61.30	56.16
$model_{10}$	74.87	80.98	75.85	77.57	60.61	62.79	70.12	62.49	59.21
$model_{11}$	77.00	82.37	75.50	77.54	60.43	56.30	73.37	64.23	56.84
$model_{12}$	88.59	83.08	77.30	79.17	64.54	64.34	75.69	66.35	59.58
$model_{13}$	88.84	84.77	73.81	77.92	68.81	59.33	72.60	67.32	57.21
$model_{14}$	70.32	67.97	59.62	64.96	59.29	37.28	63.45	58.34	30.22
$model_{15}$	84.25	81.66	74.48	69.49	67.81	62.14	70.81	68.11	60.25
$model_{16}$	83.48	82.71	70.55	78.34	68.97	60.38	72.84	68.42	61.01
$model_{17}$	93.04	88.64	79.1	81.78	67.47	64.7	78.39	68.94	61.37
ours	**94.88**	**89.34**	**81.42**	**83.71**	**70.84**	**68.67**	**83.95**	**72.98**	**66.47**

**Table 5:** Average precision (AP) (%) of the KITTI Object Detection Benchmark dataset. L, C and S in the “Sensor” column denote the LiDAR, CCD and stereo vision sensors, respectively. DPM, deformable part model.LSVM-MDPM, latent support vector machine-modified discriminative part based model; ICF, integrated channel features; BB, bounding box regression;

	Fusion	Sensor	*Cars*			*Pedestrians*			*Cyclists*		
			Easy	Moderate	Hard	Easy	Moderate	Hard	Easy	Moderate	Hard
Vote3D [59]	×	L	56.80	47.99	42.57	44.48	35.74	33.72	41.43	31.24	28.62
LSVM-MDPM [60]	×	C	68.02	56.48	44.18	47.74	39.36	35.95	35.04	27.50	26.21
SquaresICF [61]	×	C		-		57.33	44.42	40.08		-	
MDPM-un-BB [62]	×	C	71.19	62.16	48.48		-			-	
DPM-C8B1 [63]	×	S	74.33	60.99	47.16	38.96	29.03	25.61	43.49	29.04	26.20
DPM-VOC+ VP [64]	×	C	74.95	64.71	48.76	59.48	44.86	40.37	42.43	31.08	28.23
OC-DPM [65]	×	C	74.94	65.95	53.86		-			-	
AOG [66]	×	C	84.36	71.88	59.27		-			-	
SubCat [67]	×	C	84.14	75.46	59.71	54.67	42.34	37.95		-	
DA-DPM [68]	×	C		-		56.36	45.51	41.08		-	
Faster R-CNN [34]	×	C	86.71	81.84	71.12	78.86	65.90	61.18	72.26	63.35	55.90
FilteredICF [69]	×	C		-		61.14	53.98	49.29		-	
pAUCEnsT [70]	×	C		-		65.26	54.49	48.60	51.62	38.03	33.38
3DVP [71]	×	C	87.46	75.77	65.38		-			-	
Regionlets [72]	×	C	84.75	76.45	59.70	73.14	61.15	55.21	70.41	58.72	51.83
uickitti	×	C	90.83	89.23	79.46	83.49	**71.84**	67.00	78.40	70.90	62.54
Fusion-DPM [73]	Decision	L + C		-		59.51	46.67	42.05		-	
MV-RGBD-RF [74]	Early	L + C	76.40	69.92	57.47	73.30	56.59	49.63	52.97	42.61	37.42
3DOP [58]	Early	S + C	93.04	88.64	79.10	81.78	67.47	64.70	78.39	68.94	61.37
Ours (CCD)	×	C	88.84	84.77	73.81	77.92	68.81	59.33	72.60	67.32	57.21
Ours (LiDAR)	×	L	70.32	67.97	59.62	64.96	59.29	37.28	63.45	58.34	30.22
Ours (TBM)	Decision	L + C	84.25	81.66	74.48	69.49	67.81	62.14	70.81	68.11	60.25
Ours (CRF)	Decision	L + C	83.48	82.71	70.55	78.34	68.97	60.38	72.84	68.42	61.01
Ours	Decision	L + C	**94.88**	**89.34**	**81.42**	**83.71**	70.84	**68.67**	**83.95**	**72.98**	**66.47**
